# Prediction Model for Urinary Tract Infection in Pediatric Urological Surgery Patients

**DOI:** 10.3389/fpubh.2022.888089

**Published:** 2022-06-22

**Authors:** Yi Chen, Xiao-Hua Ge, Qun Yu, Ying Wang, Sheng-Mei Zhu, Jia-Ni Yuan, Wen Zong

**Affiliations:** ^1^Department of Pediatric Urology, Xinhua Hospital Affiliated to Shanghai Jiao Tong University School of Medicine, Shanghai, China; ^2^Department of Nursing, Xinhua Hospital Affiliated to Shanghai Jiao Tong University School of Medicine, Shanghai, China

**Keywords:** urinary tract infection, risk factors, predictive model, pediatric, urological surgery

## Abstract

**Background:**

Urinary tract infection (UTI) is a common complication in pediatric urological surgery patients and is associated with long-term sequelae, including subsequent recurrent infections and renal scarring. In this study, we aimed to explore the risk factors for UTI in pediatric urological surgery patients and construct a predictive model for UTI.

**Materials and Methods:**

A total of 2,235 pediatric patients who underwent urological surgery at a tertiary hospital between February 2019 and January 2020 were included. A multivariate logistic regression model was applied to identify the predictive factors, and a predictive model was constructed using a receiver operating characteristic curve. A multifactorial predictive model was used to categorize the risk of UTI based on the weight of the evidence.

**Results:**

A total of 341 patients with UTI were identified, which corresponded to a prevalence of 15.26% in pediatric urological surgery patients. Multivariate analysis identified six significant risk factors for UTI, including age <12.0 months, upper urinary tract disease, not using an indwelling drainage tube, hospital stay ≥10 days, administration of two or more types of antibiotics, and stent implantation. A combination of the aforementioned factors produced an area under the curve value of 88.37% for preventing UTI in pediatric urological surgery patients. A multifactorial predictive model was created based on the combination of these factors.

**Conclusions:**

The constructed multifactorial model could predict UTI risk in pediatric urological surgery patients with a relatively high predictive value.

## Introduction

Urinary tract infection (UTI) is the most common nosocomial infection, and it is reported that approximately 7% of children diagnosed with UTI present with fever in the first year of life. The prevalence of UTI in boys was higher during the first 3 months of life and then declined, whereas it was more common during the first year in girls (1). Studies reported that UTI accounted for 12.9, 19.6, and 11.3% of nosocomial infections in the United States, Europe, and China, respectively (2, 3). Recurrent infection occurred in ~8% of children who experienced a first episode of UTI (4). Among recurring cases, approximately 17.5% occurred during the first 3 months, and 53% occurred within 9–12 months following the initial infection (5). UTI in children was associated with longer hospital stays and exerted economic, family, and medical burdens (6).

Numerous studies have identified risk factors for UTI in children, including male sex, recurrence of UTI within 3 months, prenatal antibiotic exposure of mothers and infection with *Klebsiella* species, long-term catheter retention or double-J catheter retention, and bilateral double-J catheter retention (7–9). The Advanced Analytics Group of Pediatric Urology and ORC Personalized Medicine Group developed a model to predict the probability of recurring UTI associated with vesicoureteral reflux in children after an initial UTI; the model included age, sex, race, weight, systolic blood pressure percentile, dysuria, urine albumin-to-creatinine ratio, previous antibiotic exposure, and current medication (10). Identifying pediatric urological surgery patients at high risk for UTI is important. However, to date, there is no predictive model for UTI in these pediatric urological surgery patients. Therefore, the current study was undertaken to identify potential risk factors for UTI in pediatric urological surgery patients. Moreover, a multifactorial model was constructed to identify pediatric urological surgery patients at high risk for UTI. A categorical risk-scoring system was also created to assess the risk of UTI in patients belonging to each risk category.

## Materials and Methods

### Subjects

A total of 2,235 patients (weight: >6.25 kg) who underwent pediatric urological surgery at a tertiary hospital from February 2019 to January 2020 were prospectively analyzed. The exclusion criteria were as follows: children with UTI upon admission and children with intellectual disability. The study was approved by the Institutional Review Board of Xinhua Hospital (XHEC-D-2021-158). Written informed consent was obtained from the parents or guardians during the preoperative appointment. The sample size was based on the number of independent variables, and the expected incidence of UTI was defined as 10–20%. Given the retrospective cohort design, the required sample size had to ensure occurrence of at least 10 events for each predictor parameter for potential inclusion in the prediction model. Considering that the loss to follow-up was 10%, the minimum sample size was 612 patients.

### Variables

A total of 11 factors were identified based on the literature review, including sex, age, upper urinary tract disease, indwelling drainage tube, length of stay, invasive procedures, types of antibiotics used, stent implantation, catheter indwelling time, underlying diseases, and clinical outcomes. Data were collected by six primary nurses after they were trained regarding data collection processes.

### UTI Definition

UTI was diagnosed according to the 2010 Guidelines for the Diagnosis and Treatment of Common Renal Diseases in Children (11). UTI symptoms included frequent micturition, dysuria or tenderness in the lower abdomen, and percussion pain in the renal area. White blood cells in the urine test were ≥5/HP in males and ≥10/HP in females. Moreover, at least one of the following criteria had to be fulfilled: (1) gram-positive bacteria ≥104 CFU/mL, gram-negative bacteria ≥105 CFU/mL at clean midstream urine or urine retained by catheterization; (2) total bacterial colony count of ≥103 CFU/mL in urine samples obtained by suprapubic bladder puncture; (3) bacteria observed in 15 of 30 visual fields using phase-contrast microscopy in fresh urine samples centrifuged 400 times; and (4) asymptomatic bacteriuria: children with a history of endoscopy or indwelling catheterization in the past week without clinical symptoms, gram-positive bacteria ≥104 CFU/mL, and gram-negative bacteria ≥ 105 CFU/mL detected in urine (12).

### Statistical Analysis

Statistical analyses were conducted using SPSS 13.0 (SPSS Inc., Chicago, IL, United States). The baseline characteristics between patients with and without UTI were categorized as continuous or categorical variables. The *t-*test and Mann–Whitney U-test were used to assess between-group differences for continuous data exhibiting normal distribution. Categorical data between groups were analyzed using the chi-square test. Univariate analysis was then performed to identify the risk factors, which were subsequently subjected to the multivariate logistic regression model, using α = 0.05 and β = 0.10, respectively. Multivariate logistic regression was performed to identify the risk factors for UTI after the continuous adjusted variables underwent multivariate analysis. The importance of each specific factor was obtained from the regression coefficients to calculate the weight of the evidence, and the categorical risk-scoring system was constructed. Scores were obtained for numbers rounded to the nearest unit. The scores were then combined to construct a prognostic model, and the receiver operating characteristic (ROC) curve was constructed to assess multifactorial risk for UTI, while the risk accuracy was analyzed and the area under the curve (AUC) was applied. The inspection level was two-sided. Statistical significance was set at *P* < 0.05 for the identified risk factors.

## Results

### Baseline Characteristics

A total of 2,360 pediatric urological surgery patients were recruited, and 125 patients were excluded owing to incomplete data (*n* = 112), hospitalization time ≤ 24 h (*n* = 11), and patients with intellectual disability (*n* = 2). The remaining 2,235 patients (mean age: 42.47 months; boys: 1,898; girls: 337) were included. A total of 341 UTI cases (15.26%) were observed. Table 1 summarizes the characteristics of patients with and without UTI.

**Table 1:** Baseline characteristics of participants.

**Variable**	**Overall**	**UTI status**			
		**Non-UTI (1894)**	**UTI (341)**	**OR (95% CI)**	**P-value**
Sex (girls)	337 (15.08)	220 (11.62)	117 (34.31)	3.975 (3.052–5.176)	<0.001
Age (months)	42.47 (39.82)	46.32 (39.96)	21.05 (31.38)	0.975 (0.971–0.980)	<0.001
Age (≥12 months)	1700 (76.06)	1546 (81.63)	154 (45.16)	0.185 (0.145–0.236)	<0.001
Upper urinary tract disease	951 (42.55)	628 (33.16)	323 (94.72)	36.171 (22.289–58.701)	<0.001
Indwelling drainage tube (yes)	479 (21.43)	294 (15.52)	185 (54.25)	6.454 (5.043–8.259)	<0.001
Length of stay (days)	6.64 (4.94)	6.10 (4.59)	9.62 (5.74)	1.133 (1.108–1.158)	<0.001
Length of stay (≥10 days)	543 (24.30)	381 (20.12)	162 (47.51)	3.594 (2.826–4.571)	<0.001
Invasion procedures (yes)	2101 (94.00)	1776 (93.77)	325 (95.31)	1.349 (0.790–2.304)	0.273
Type of antibiotics used	0.82 (1.11)	0.62 (0.91)	1.97 (1.34)	2.813 (2.502–3.164)	<0.001
Type of antibiotics used (≥2)	660 (29.53)	429 (22.65)	231 (67.74)	7.170 (5.577–9.218)	<0.001
Implantation of stents (yes)	614 (27.47)	398 (21.01)	216 (63.34)	6.495 (5.076–8.310)	<0.001
Catheter indwelling time (days)	2.97 (3.96)	2.76 (3.98)	4.19 (3.62)	1.084 (1.056–1.113)	<0.001
Catheter indwelling time (≥5 days)	684 (30.60)	509 (26.87)	175 (51.32)	2.869 (2.267–3.630)	<0.001
Underlying disease (yes)	919 (41.12)	692 (36.54)	227 (66.57)	3.459 (2.711–4.413)	<0.001
Clinical outcome (cure)	2039 (91.23)	1728 (91.24)	311 (91.20)	0.996 (0.663–1.496)	0.984

### Univariate Analysis

There were significant differences between the UTI and non-UTI groups for sex ([odds ratio] OR: 3.975; 95% [confidence interval] CI: 3.052–5.176; *P* < 0.001), age ≥12 months (OR: 0.185; 95% CI: 0.145–0.236; *P* < 0.001), upper urinary tract disease (OR: 36.171; 95% CI: 22.289–58.701; *P* < 0.001), indwelling drainage tube (OR: 6.454; 95% CI: 5.043–8.259; *P* < 0.001), hospital stay ≥10 days (OR: 3.594; 95% CI: 2.826–4.571; *P* < 0.001), two or more types of antibiotics used (OR: 7.170; 95% CI: 5.577–9.218; *P* < 0.001), stent implantation (OR: 6.495; 95% CI: 5.076–8.310; *P* < 0.001), catheter indwelling time ≥5 days (OR: 2.869; 95% CI: 2.267–3.630; *P* < 0.001), and underlying disease (OR: 3.459; 95% CI: 2.711–4.413; *P* < 0.001). However, there were no significant differences between the UTI and non-UTI groups for invasive procedures (OR: 1.349; 95% CI: 0.790–2.304; *P* = 0.273) and clinical outcome (OR: 0.996; 95% CI: 0.663–1.496; *P* = 0.984).

### Multivariate Analysis

**Figure 1:**
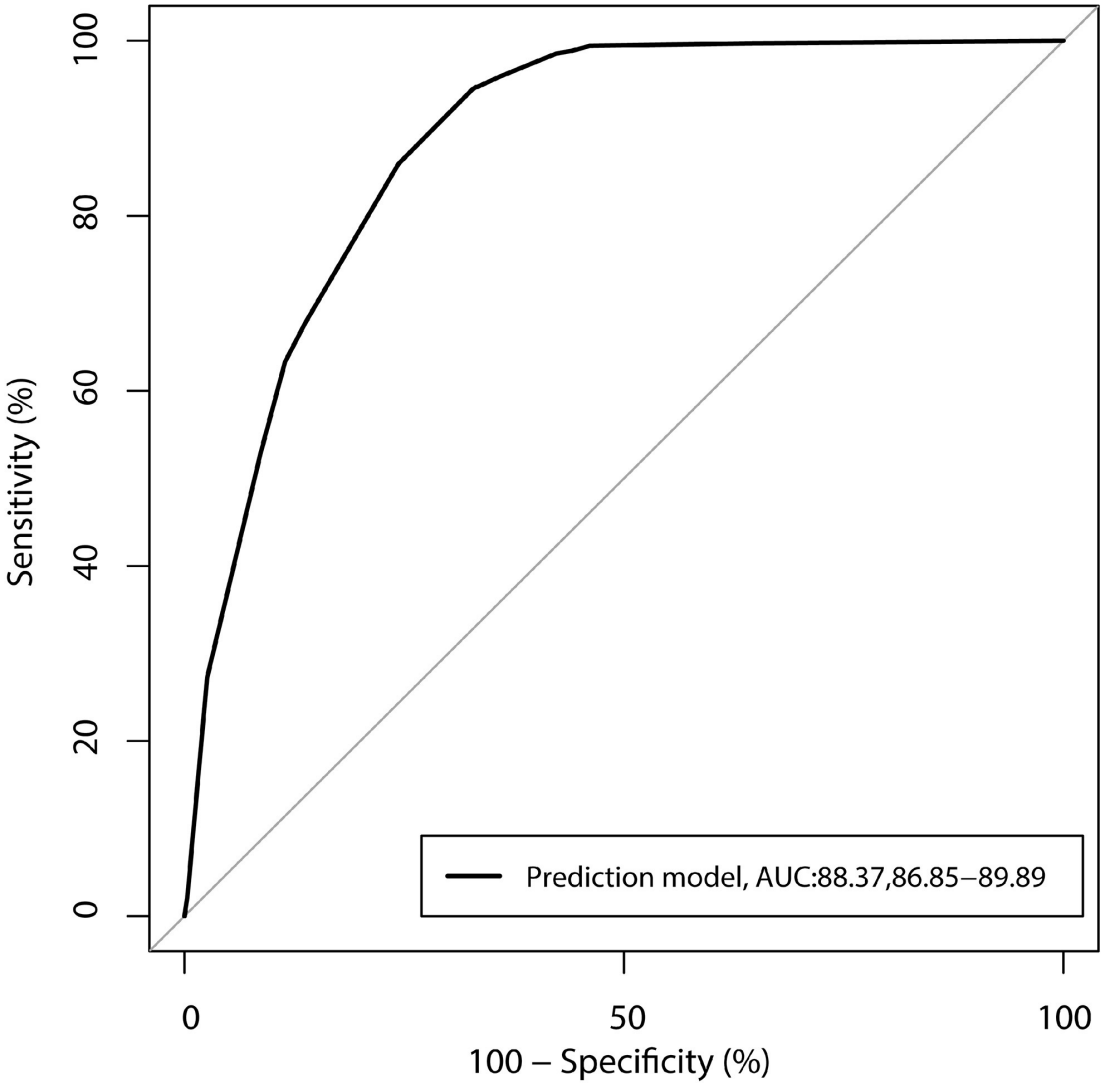
Receiver operating characteristic curve for the risk of urinary tract infection, including the six-component risk factor model (area under the curve = 88.37%).

According to the inclusion and exclusion criteria, invasive procedures and clinical outcomes were removed from the model. Multiple stepwise regression was performed, and the results are summarized in Table 2. We considered age <12.0 months (OR: 2.100; 95% CI: 1.573–2.804; *P* < 0.001), upper urinary tract disease (OR: 21.611; 95% CI: 12.104–38.585; *P* < 0.001), not using an indwelling drainage tube (OR: 1.680; 95% CI: 1.174–2.405; *P* = 0.005), hospital stay ≥10 days (OR: 1.722; 95% CI: 1.234–2.403; *P* = 0.001), two or more types of antibiotics used (OR: 4.384; 95% CI: 3.027–6.348; *P* < 0.001), and stent implantation (OR: 1.440; 95% CI: 1.052–1.972; *P* = 0.023) were associated with an increased risk of UTI. These six risk factors were combined to construct the ROC curve to determine the risk of UTI, and the AUC was 88.37% (Figure 1).

**Table 2:** Scores of particular risk factors in the predictive model for UTI.

**Variable**	**β**	**OR(95% CI)**	**P–value**	**Score**
Age				
<12 months	0.742	2.100 (1.573–2.804)	<0.001	1
≥12 months	0	1		0
Upper urinary tract disease				
No	0	1		0
Yes	3.073	21.611 (12.104–38.585)	<0.001	15
Indwelling drainage tube				
No	0.519	1.680 (1.174–2.405)	0.005	1
Yes	0	1		0
Length of stay				
<10 days	0	1		0
≥10 days	0.543	1.722 (1.234–2.403)	0.001	1
Type of antibiotics used				
<2	0	1		0
≥2	1.478	4.384 (3.027–6.348)	<0.001	3
Implantation of stents				
No	0	1		0
Yes	0.365	1.440 (1.052–1.972)	0.023	1

### Establishment of the Risk Scoring System

**Figure 2:**
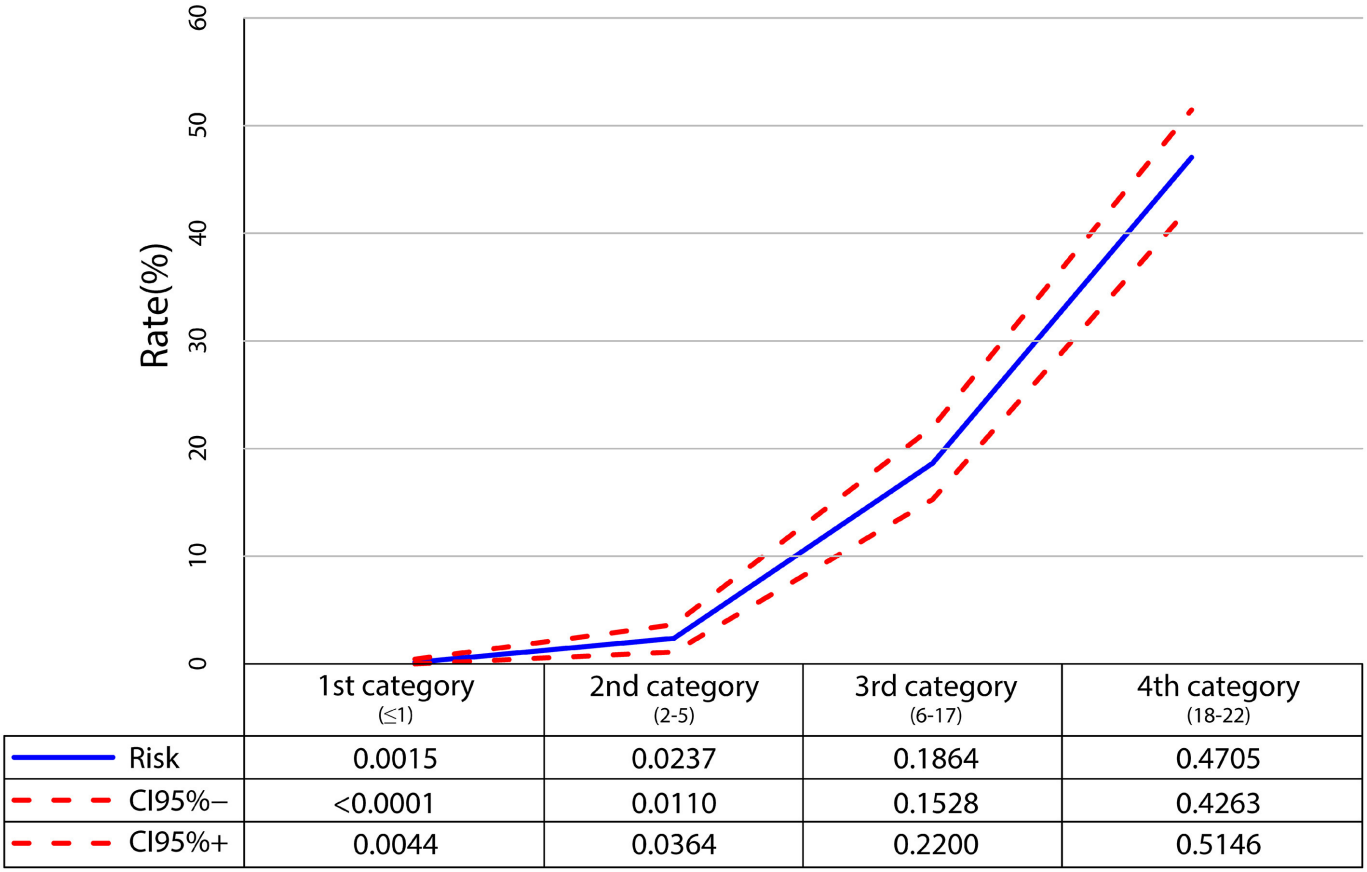
Probability of developing urinary tract infection for particular risk categories.

The risk score was obtained based on the multivariate logistic regression model, and the details of the scoring system for the six risk factors are listed in Table 2. The four categories for predicting UTI risk are shown in Figure 2, which are identified by summing the scores. Results based on the scores are listed as follows: 680 children exhibited low risk (score: ≤ 1, mean risk: 0.15% [95% CI: <0.01–0.44%]); 549 children were moderately low risk (score: 2–5, mean risk: 2.37% [95% CI: 1.10–3.64%]); 515 children were moderately high risk (score: 6–17; mean risk: 18.64% [95% CI: 15.28%−22.00%]), and 491 children were high risk (score: 18–22, mean risk: 47.05% [95% CI: 42.63–51.46%]).

## Discussion

We aimed to construct a multifactorial predictive model for the development of UTI in pediatric urological surgery patients, which could be applied as a simple predictive tool for identifying pediatric urological surgery patients at high risk of developing UTI and thereby applying preventive strategies for the modifiable factors. This study was the first predictive model for UTI in pediatric urological surgery patients in China, as previous studies performed in China focused on identification of the risk factors (13–15).

We observed that younger children exhibited an increased risk for UTI. This could be because the organs and immune systems were not fully developed, and therefore the ability to resist external pathogens was compromised. Similar to the longer hospital stay, the risk for UTI was significantly increased due to various hospital-acquired pathogenic bacteria. Urinary tract disease in patients was associated with urinary obstruction because the bacteria could not be flushed by urine; therefore, large numbers of bacteria were deposited in the urine, causing increased pressure above the obstruction. These conditions could create lower local resistance of the mucous membrane due to poor blood supply rather than the UTI itself (16). Moreover, in the predictive model, children with upper urinary tract disease with a heavy weight could be considered a main risk factor for UTI in pediatric urological surgery patients.

We found that the use of an indwelling drainage tube was a protective factor for UTI, which was inconsistent with prior studies (9, 17, 18). Wang et al. performed a retrospective study of 107 infants and young children and found that indwelling drainage tube, male, and double-J catheter retention were independent risk factors for UTI (9). A retrospective study conducted by Kitano et al. showed that the indwelling drainage tube, hydronephrosis, and renal stones were associated with an increased risk for UTI (17). Fukuoka et al. found that the increased number of days an indwelling drainage tube was used was associated with an increased risk for UTI in children at PICU (18). This may be because upon insertion of the drainage tube, bacteria could adhere to the tube wall and generate bacterial biofilms on the tube surface. The same reason could explain the use of stent implantation as a risk factor for UTI. Microscopic analysis of the bacterial biofilms revealed a honeycomb structure, which was difficult for antibiotics to infiltrate, and could result in multidrug resistance through repeated antibiotic use (19). The use of an indwelling drainage tube, which could reduce the risk for UTI, could be explained by the following: the underlying effect of an indwelling drainage tube was balanced by other characteristics; the bundle compliance to eliminate the dependent loop in the drainage tube through targeted rounds ensured that the appropriate use of the catheter could reduce the risk for UTI (20); the appropriate indication for the insertion of the drainage tube, standardization of care for the indwelling drainage tube, appropriate management of leaking urinary catheters, urinary catheter days and removal were combined in daily rounds, and the prolonged use of an indwelling drainage tube should be carefully monitored to improve the progression of UTI (21).

The predictive model for UTI in pediatric urological surgery patients was constructed, and a total of six risk factors were identified in the multivariate analysis. The AUC of our model was 88.37%, which was relatively high, and this model could be simply applied in clinical practice. Moreover, these factors could be monitored and results of the scoring system could be obtained daily; children at high risk could be monitored and intervention strategies implemented to prevent the progression of UTI. Therefore, the training for risk assessment of pediatric urological surgery patients should be provided to nurses (22, 23).

This study has several limitations. First, there are several diseases requiring urological surgery, which could affect the progression of UTI. Second, urinary tract disease in patients was the strongest risk factor after adjusting for other factors, which might have induced uncontrolled factors. Last, the predictive model of our study lacks external validation, and the scalability and interoperability of our predictive model should be verified at other centers and clinical settings.

## Conclusions

We constructed a predictive model for UTI in pediatric urological surgery patients. Six risk factors were entered into the model, including age <12.0 months, upper urinary tract disease, not using an indwelling drainage tube, hospital stay ≥10 days, two or more types of antibiotics used, and stent implantation. Moreover, the predictive value of this model was relatively high. Therefore, clinical management adjusted based on the prediction model is strongly warranted. Further large-scale prospective cohort studies at other centers and clinical settings should be performed to validate the predictive value of this model.

## Data Availability Statement

The raw data supporting the conclusions of this article will be made available by the authors, without undue reservation.

## Ethics Statement

The studies involving human participants were reviewed and approved by Institutional Review Board of Xinhua Hospital. The patients/participants provided their written informed consent to participate in this study. Written informed consent was obtained from the individual(s) for the publication of any potentially identifiable images or data included in this article.

## Author Contributions

YC conceived and designed the experiments. YW, S-MZ, J-NY, and WZ performed the experiments. YC analyzed the data and wrote the paper. X-HG and QY contributed to reagents, materials, and analysis tools. All authors have read and approved the final version of this manuscript.

## Funding

This study was funded by Science and Technology Fund project of Shanghai Jiaotong University (Jyhz1920). The funder played no role in the study design, data collection and analysis, decision to publish, or preparation of the manuscript.

## Conflict of Interest

The authors declare that the research was conducted in the absence of any commercial or financial relationships that could be construed as a potential conflict of interest.

## Publisher's Note

All claims expressed in this article are solely those of the authors and do not necessarily represent those of their affiliated organizations, or those of the publisher, the editors and the reviewers. Any product that may be evaluated in this article, or claim that may be made by its manufacturer, is not guaranteed or endorsed by the publisher.
